# Splenic Abscess: A Rare Complication of Bacterial Pneumonia

**DOI:** 10.7759/cureus.35432

**Published:** 2023-02-24

**Authors:** Modupeoluwa Owolabi, Ruhma Ali, Amy Paige, Alaa Muhanna, Jihad Slim

**Affiliations:** 1 Internal Medicine, Saint Michael's Medical Center, Newark, USA; 2 Pulmonary and Critical Care Medicine, Saint Michael's Medical Center, Newark, USA; 3 Infectious Diseases, Saint Michael's Medical Center, Newark, USA

**Keywords:** management of splenic abscess, bacterial pneumonia, contiguous spread, pneumonia complication, splenic abscess

## Abstract

Splenic abscess is a rare condition with potentially life-threatening evolution. Hematogenous spread is the most common cause of splenic abscess. Contiguous spread after bacterial pneumonia has rarely been reported in the literature. Early diagnosis can be made by a combination of imaging modalities and clinical features. The successful management of splenic abscess includes timely medical therapy, computed tomography (CT)-guided percutaneous aspiration, and splenectomy. In this report, we discuss a rare case of splenic abscess after hospitalization for bacterial pneumonia. The aim of this case report is to raise awareness about this rare complication so that prompt and appropriate management can be quickly performed to prevent severe outcomes.

## Introduction

Splenic abscess is an uncommon entity with a reported incidence of 0.05%-0.07% [1]. Splenic abscess generally occurs in patients with splenic infarcts, neoplasia, trauma, or immunodeficiency and is often due to the hematogenous spread of infections [2]. Bacterial pneumonia complication can arise even with appropriate antibiotic management; however, contiguous spread to the spleen is a very rare occurrence. We present the case of a 63-year-old male who developed a splenic abscess after hospitalization for bacterial pneumonia. This case is being reported to increase awareness of such a rare complication after hospitalization and the importance of prompt diagnosis and antibiotic management to improve outcome.

## Case presentation

A 63-year-old male with a past medical history of hypertension came to the emergency department with complaints of generalized weakness and fatigue for one day. The patient states that he is homeless. The patient also complained of shortness of breath (SOB), fever, dry cough, and a loss of appetite for the same duration of time. The patient stated that he received three doses of BNT162b2 vaccination, but he did not receive his flu vaccination. The patient did not have any dizziness, syncope, loss of consciousness, trauma, vertigo, blurry vision, sore throat, nausea, vomiting, abdominal pain, diarrhea, constipation, chest pain, palpitation, dysuria, chills, night sweats, and weight loss. The patient stated that he drinks alcohol occasionally but has no history of smoking or illicit drug use.

**Figure 1:**
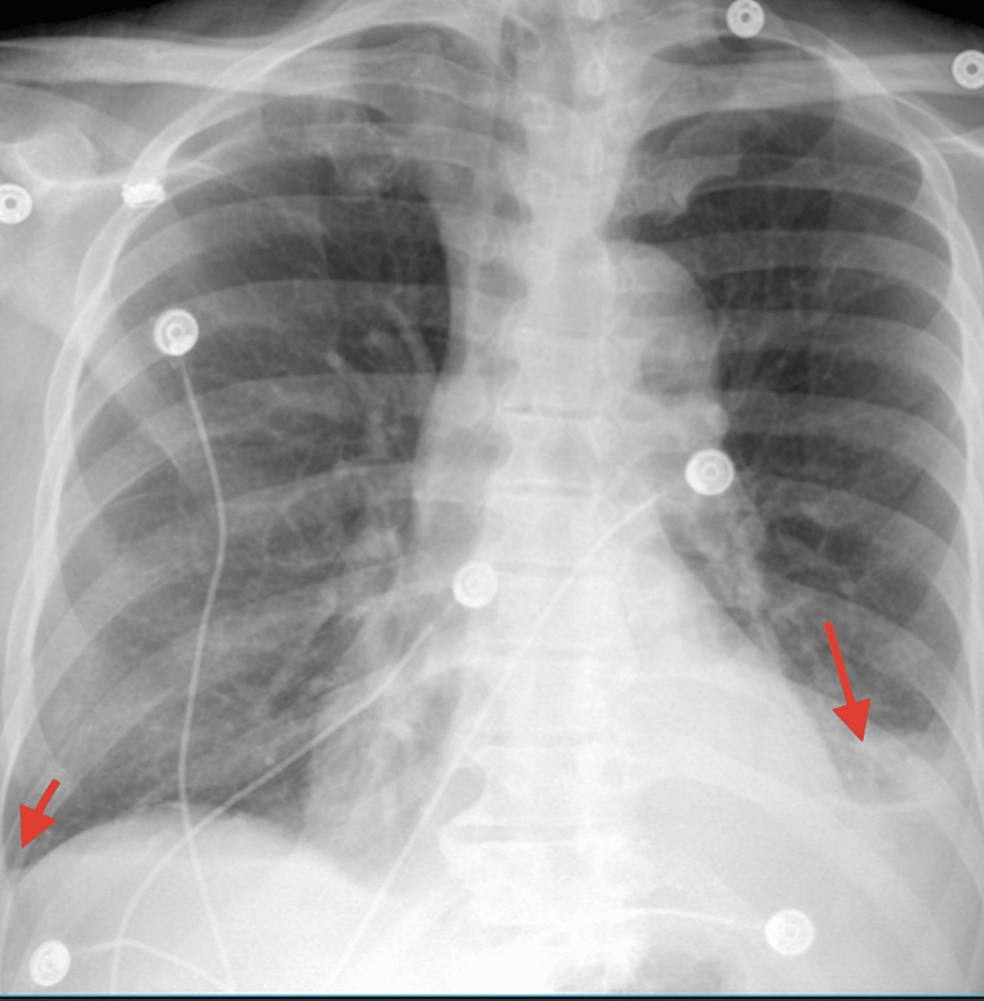
Red arrows show bilateral pleural effusions with possible underlying infiltrates in the left lung base

On admission, his body temperature was 103.3°F, blood pressure 95/49 mmHg, heart rate 110 beats/minute, respiratory rate 24 breaths/minute, and saturation at 95% on room air. The patient was alert and oriented to person and place but not to time. Physical examination was notable for mild rales in the left lower lobe and bilateral ankle edema. The patient was unable to walk due to weakness; however, strength was 5/5 in both upper and lower extremities, and sensation was intact in the upper and lower extremities; the rest of the physical examination was unremarkable. The patient's Wells score for pulmonary embolism was 4.5, indicating a moderate risk for pulmonary embolism. Chest X-ray showed bilateral pleural effusion with an underlying infiltrate in the left lung base as shown in Figure 1.

**Figure 2:**
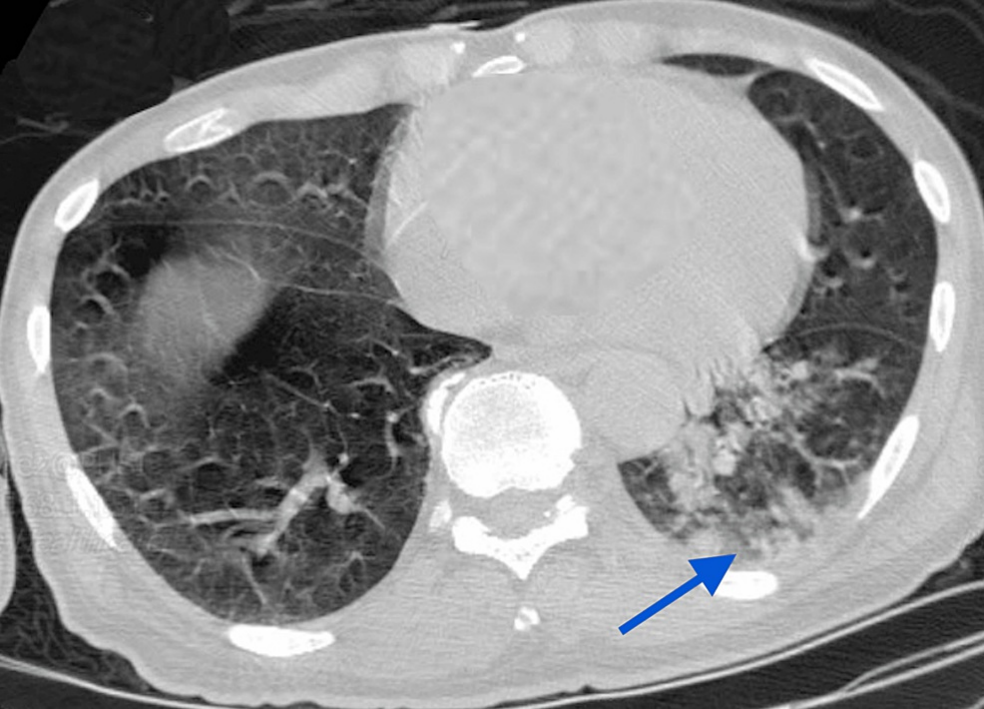
The blue arrow indicates left lower lobe parenchymal consolidation with air bronchogram

Computed tomography (CT) scan of the chest without contrast showed left lower lobe parenchymal consolidation with air bronchogram as shown in Figure 2. Ventilation perfusion scan showed low probability for pulmonary embolism. Bilateral venous Doppler ultrasound of the lower extremities showed no evidence for acute deep vein thrombosis. Electrocardiogram showed normal sinus rhythm with left anterior fascicular block and left ventricular hypertrophy. SARS-CoV-2 reverse transcription-polymerase chain reaction (RT-PCR) and molecular testing for influenza A and B were all negative.

All inflammatory markers were elevated as indicated in Table 1. *Legionella* antigen, *Streptococcus pneumoniae* antigen, and respiratory pathogen panel were negative. Thoracentesis was not performed because after bedside ultrasound was performed, the volume of fluid was not sufficient to be drained. The patient was started on doxycycline and ceftriaxone for the coverage of community-acquired pneumonia (CAP), which was later switched to oral levofloxacin. Blood culture and sputum culture were negative for any microorganism. During the course of hospital stay, the patient continued to have persistently low-grade fever.

**Table 1:** Initial laboratory data HS, high-sensitivity; LDH, lactate dehydrogenase; CRP, C-reactive protein

Laboratory Parameters	Values	Reference Range
Sodium	134	136-145 mmol/L
Potassium	4.5	3.5-5.3 mmol/L
Chloride	97	98-110 mmol/L
Blood Urea Nitrogen (BUN)	88	6-24 mg/dL
Creatinine	2	0.6-1.2 mg/dL
Aspartate Transaminase (AST)	99	10-36 U/L
Alanine Transaminase (ALT)	41	9-46 U/L
White Blood Cell (WBC)	5.90	4.4-11 × 10^3^/μL
Hemoglobin	12.9	13.5-17.5 g/dL
Platelets	76	150-450 × 10^3^/μL
D-Dimer	25,147	0.0-500 ng/ml
HS Troponin I	8	0-76 ng/L
Procalcitonin	6.5	0-0.50 ng/ml
LDH	476	122-222 U/L
CRP	5.5	0.0-0.8 mg/dL
Ferritin	1,650	24-336 ng/ml

**Figure 3:**
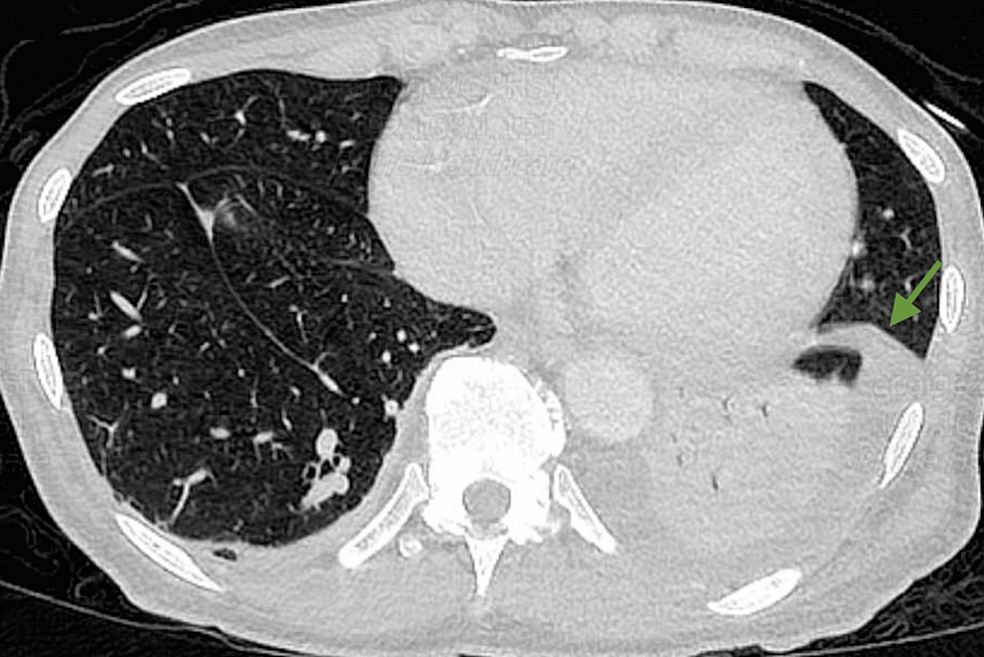
The green arrow indicates an ovoid hypodense area in the lateral basal segment of the left lower lobe consistent with a lung abscess

**Figure 4:**
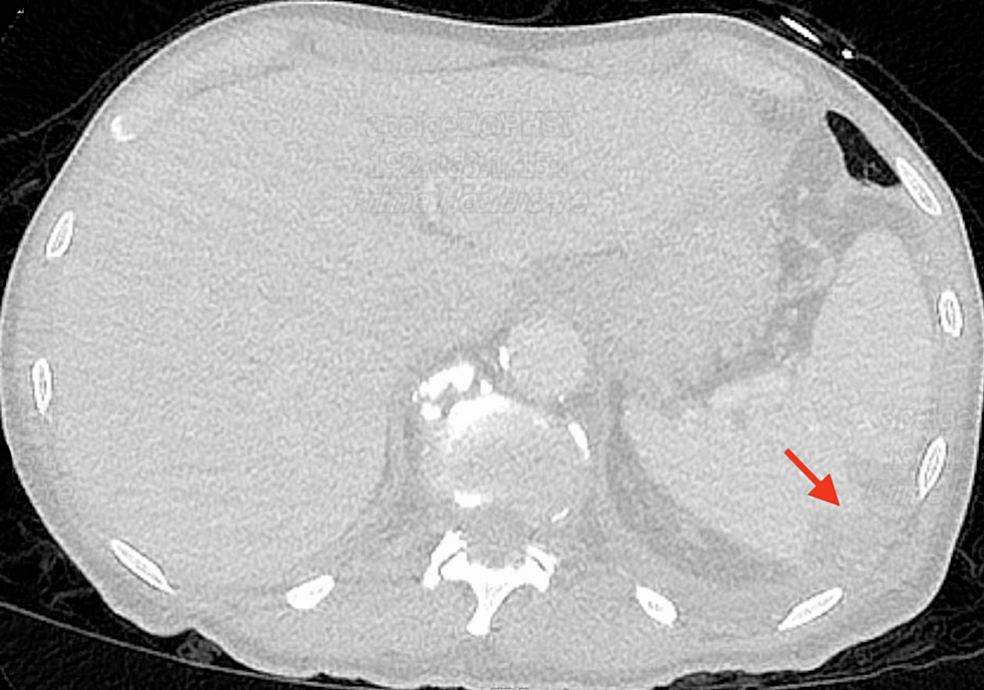
The red arrow indicates irregularly shaped low-attenuation lesion in the lateral aspect of the spleen measuring up to 3.5 cm, indicating a possible subscapular intrasplenic abscess (day 17)

**Figure 5:**
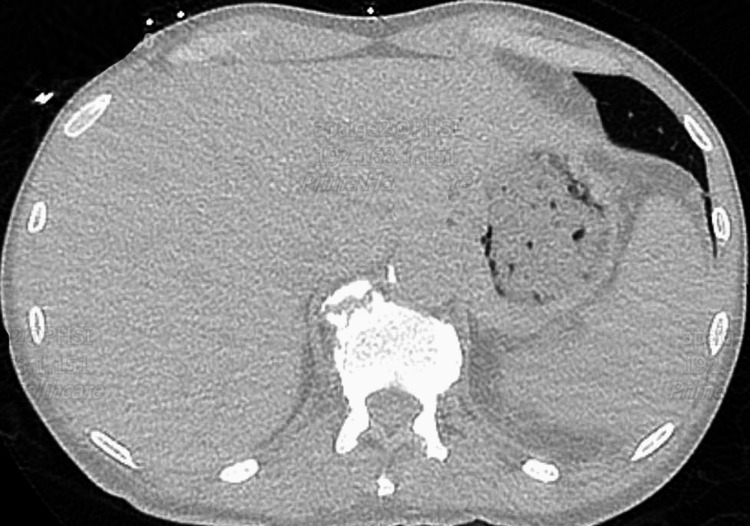
No splenic abscess (day 22)

On day 17, a repeat CT scan of the chest with contrast showed a 2 cm ovoid hypodense area in the lateral basal segment of the left lower lobe consistent with a lung abscess. A crescent-shaped hypodensity in the spleen measuring 3 × 1.5 cm, indicating a possible subscapular intrasplenic abscess, was also noted (Figure 3). CT of the abdomen and pelvis with IV contrast showed irregularly shaped low-attenuation lesion in the lateral aspect of the spleen measuring up to 3.5 cm (Figure 4). Transthoracic echocardiogram (TTE) and transesophageal echocardiogram (TEE) were negative for valvular vegetations. Interferon gamma release assay test was performed due to a high risk factor (homeless), and the result was negative. The encapsulated splenic abscess was inadequate for drainage because the cavity was poorly defined. Taking the new imaging findings into consideration, antibiotic coverage with vancomycin and Zosyn was started. Repeat CT scan on day 22 showed a decrease in the size of the lung abscess and no splenic abscess as shown in Figure 5. The patient improved clinically with no further febrile episodes and was discharged with outpatient follow-up.

## Discussion

Splenic abscess is an uncommon infection that typically results from hematogenous spread. Splenic abscess can be caused by *Streptococcus*, *Staphylococcus*, *Escherichia coli*, and *Salmonella* [3]. Other causes of splenic abscess include traumatic splenic injuries and contiguous invasion of the spleen by intra-abdominal abscesses [4]; however, splenic abscess caused by contiguous spread after bacterial pneumonia has rarely been reported. In this report, We describe a unique case of splenic abscess that occurred in a patient with bacterial pneumonia via contiguous spread. Pneumonia is a bacterial, viral, or fungal infection that can affect one or both lungs. The infection causes the air sac to become inflamed and fill up with fluid or pus [5].

In the United States, about four million patients are treated yearly for community-acquired pneumonia (CAP). Pneumonia remains the most common cause of hospital admissions in the United States. Despite the widespread adherence to treatment recommendations and the availability of resources, pneumonia remains the eighth leading cause of death in the United States; nevertheless, many healthcare providers underestimate the mortality associated with the infection [6]. The financial burden associated with CAP yearly in the United States remains significant at about $17 billion annually [6]. The mode of transmission of pneumonia is mainly via the micro-aspiration of respiratory droplet; less frequent spread can also occur via touch transfer between surfaces that have the microorganisms and touching of the nose or mouth [5]. *Streptococcus pneumoniae* and *Klebsiella pneumoniae* are frequent causes of community-acquired respiratory infections in adults [7]. The risk factors for pneumonia include old age and medical conditions such as lung diseases, heart diseases, weakened immune system or neurologic conditions that affect swallowing, and predisposition to aspiration pneumonia. Health behaviors such as cigarette smoking and alcohol and drug abuse can also increase the possibilities of aspiration, which can predispose to pneumonia [5].

In this case report, the patient's risk factor was his history of alcohol use. The clinical presentation of pneumonia can range from mild symptoms such as cough (productive or nonproductive), fever, chills, sweating, SOB, decreased appetite, fatigue, low energy, nausea, and vomiting to severe symptoms needing hospitalization such as pleuritic chest pain, tachycardia, and confusion [8]. The patient in this case presented with a nonproductive cough, fever, fatigue, weakness, SOB, decreased appetite, and tachycardia. The diagnostic modality for pneumonia includes a patient's health history, physical examination, and symptoms and some diagnostic tests such as blood tests that check for inflammatory markers such as procalcitonin, chest X-ray, blood cultures, sputum culture, pulse oximetry, CT scan of the chest, bronchoscopy, and pleural fluid culture. In this case, the patient's history, physical examination, elevated procalcitonin, and CT scan of the chest findings lead to the diagnosis of bacterial pneumonia.

Procalcitonin has been shown to have great differentiating significance for distinguishing between bacterial and viral infection; thus, it helped with the diagnosis of this patient's bacterial pneumonia; in addition, the turnaround time for results is within a couple of hours. The procalcitonin result has greater than 65% accuracy in distinguishing between patients who have viral and bacterial etiology in patients with community-acquired pneumonia [9]. Splenic abscess can be diagnosed by imaging modalities such as ultrasound, CT, and MRI. Splenic abscess is usually managed by a combination of splenectomy and antibiotic therapy. Successful therapeutic results have also been obtained via CT-guided aspiration. Izumikawa et al. reported a case where vancomycin therapy was sufficient for treatment [10]. In this case, the patient improved markedly with antibiotic therapy, which further supports the hypothesis that the splenic abscess was caused by direct organism invasion. The mortality rate in patients with splenic abscess is very high [11]. Patients with immunodeficiency and multiple abscesses are suggested to have a poor prognosis and increased mortality. Prompt diagnosis and treatment are required to improve outcomes in patients with high index of suspicion.

## Conclusions

Patients with pneumonia can present with complications such as acute respiratory distress syndrome, lung abscess, respiratory failure, sepsis, and splenic abscess like the case presented in this report. It is essential that providers recognize the complications of pneumonia early, in order to be able to manage the patient appropriately and decrease morbidity and mortality. Patients with splenic abscess, from contiguous spread, can be managed with antibiotics based on the type of pneumonia. Special attention should be given to elderly patients and patients with underlying health conditions to decrease the likelihood of severe outcomes.
